# 

**Published:** 1894-08-25

**Authors:** 


**Notices of Births, Deaths, and Marriages** are inserted in this column at a charge of 2s. 6d. for an announcement not exceeding four lines.

**Notice to Advertisers and Subscribers.**

All Advertisements, Orders for Copies of The Hospital, and Business Communications should be addressed to **The Publisher, NOT TO THE EDITOR**, The Hospital, 428, Strand, W.C.

The Cost of Subscription, post free, is as follows:—Per week, 2½d.; per quarter, 2s. 8d.; per half-year, 5s. 3d.; per annum, 10s. 6d. Foreign:—Per Annum, 12s. 8d.; payable, in all cases, in advance.

### NOTICE TO CORRESPONDENTS.

All MS., Letters, Books for Review, and other matters intended for the Editor should be addressed The Editor, The Lodge, Porchester Square, London, W.

The Editor cannot undertake to return rejected MS., even when accompanied by stamped directed envelope.

**"Burdett's Hospital Annual," 1894.**

Fifth year of publication. 900 pp. Boards, gilt lettering. A most complete guide to British, Colonial, Indian, and American Hospitals, Dispensaries, Nursing and Convalescent Institutions, and Asylums. Price 5s. Published by The Scientific Press (Limited), 428, Strand, W.C.

**NOTICE.—The Index for Vol. XV. (ending March 31st, 1894) is on sale at the Office of this Journal, price 2½d., post free.**

### Scraps and Gleanings.

The Duke of York has consented to become patron of the Seaside Convalescent Home, Seaford, Sussex.

A proposed Joint Infectious Diseases Hospital for the town and district is under discussion at Tiverton.

The annual meeting on behalf of the Hertfordshire Seaside Convalescent Home was held in the gardens at Childwick Bury this month.

The expenses of the new ward for the Derbyshire Royal Infirmary are to be defrayed by Mr. G. H. Strutt, and the ward is to be called after the late Miss Susan Strutt.

Hospital Saturday collections at Shrewsbury amount this year to £127 9s. 7d. A considerable item in the receipts was the sum obtained from the Quarry Concert.

A new infirmary is to be erected at Bowes Manor, Bowes Park Islington. The cost will be about £125,000, and the buildings will take about two years to construct.

The Rev. C. G. K. Gillespie, of St. Andrews, Boston, has been appointed delegate of the Church Sanitary Association for the forthcoming Congress of Hygiene at Budapest.

A collection in aid of the Devon and Exeter Hospital is to be made on the first Friday in September at Exeter. The committee are discussing the advisability of adding a new wing to the hospital.

The Duke of Norfolk has given £5,000 to the Sheffield Public Hospital towards the fund for rebuilding the institution. Fifty thousand pounds is the sum required, £17,000 of which is already promised.

Two cases of genuine leprosy have been ascertained in the clinical department of the University of Breslau. The sanitary authorities have taken careful measures to prevent the disease from spreading.

The new out-patients' department and nurses' quarters of the Central London Throat and Ear Hospital in Gray's Inn Road are to be commenced at once. The plans adopted are by Mr. E. Turner, F.R.I.B.A.

The Committee of the Royston Cottage Hospital in Hertfordshire report the financial condition of the institution as far from satisfactory, and it has been again necessary to borrow from the original Building Fund.

This month the eighth International Ophthalmological Congress was opened at Edinburgh. Technical communications on the extraction of cataract were submitted by Professor Snellen, of Utrecht, and Professor Knapp, of New York.

The eighth annual musical festival and demonstration of Friendly Societies took place last month at Normanton on behalf of the Leeds Infirmary and the Clayton Hospital. The result of the demonstration reached a total of £44 0s. 5d.

The new wing has been opened at Lloyd's Cottage Hospital, Bridlington. A bazaar was held in the day wards, which were fitted up with stalls for the occasion, and a quantity of fancy articles were disposed of for the benefit of the building fund.

Two diamond rings were found in one of the offertory bags of Holy Trinity Church, Twickenham, on Hospital Sunday. The donor is unknown, the rings being wrapped in paper with instructions that they should be sold for the benefit of St. John's Hospital.

We are requested to state that the Convalescent Home, Folkestone, mentioned in "Construction Notes" in The Hospital of August 11th, is not the St. Andrew's Convalescent Home which was built some years ago on the east cliff, and has accommodation for 150 patients.

A new Convalescent Home for the Working Men's Club and Institute Union has been opened at Pegwell Bay, near Ramsgate. The Home is the gift of Mr. Passmore Edwards, and contains about thirty rooms. The affiliated clubmen will be entitled to free residence during convalescence.

A gala and sports were held at Deighton, Shreepridge, this year in aid of the Huddersfield Infirmary. The entertainment included horse-leaping, steeplechase, and an amateurs' trotting handicap. After the sports were over the gala concluded with a good display of fireworks in the evening.

Bedford was besieged on Hospital Saturday by a Red Cross Army, which enlisted itself in aid of the General Infirmary. A house-to-house collection was made in the morning, and in the afternoon the town band paraded the streets, and collected *en route*. The total collections amounted to about £300.

In accordance with the resolution of the Court of Common Council respecting the precautions against cholera the medical assistant to the Medical Officer of Health for the Port of London is to be stationed at Sheerness. In conjunction with the Customs he will medically inspect all vessels entering the Medway.

A friend of the late Mr. Cairnes, of Dublin, has offered £500 towards the new wing to the Rotunda Hospital, on condition that the new building is called "The Thomas Plunket Cairnes Wing of the Rotunda Hospital," and that other subscriptions augment his gift to £700. The Board has gratefully accepted his offer.

The committee of the Scarlet Fever Hospital at Birmingham have decided on extensive improvements and additions. It is suggested to erect two pavilions, each accommodating eighty patients, and plans are under discussion for a receiving and discharging block and an isolation ward for six beds. The cost of the proposed works is £38,000.

It was stated at the annual general meeting of the subscribers to the Children's Hospital and Dispensary at Shirley that there has been a very large falling off this year in receipts from entertainments, and the committee earnestly solicit additional subscriptions. The hospital accounts, however, show the institution to be still free from debt.

A long discussion took place at the quarterly meeting of the Forfar Infirmary directors concerning the cases of scarlet fever under treatment in the institution. It was decided to hold a special general meeting to consider the erection of a separate wash-house for the benefit of infectious cases. The treasurer reported the funds in hand to be £210 12s. 6d.

The first annual meeting of the Hull and East Riding Convalescent Home has been held this summer in the form of a garden party in the grounds of the Home at Withernsea. The Chairman, in his report, pointed out that by payment of a guinea to the treasurer anyone could recommend a candidate for the benefits of the Home, with a three weeks' residence at the seaside.

### BOOKS RECEIVED.

Crosby Lockwood and Son.

"The Sanitary Arrangement of Dwelling Houses." By A. J. Wallis-Taylor, C.E.

**Periodicals and Pamphlets.** — Medical Magazine, Medical Record, The Scalpel, Leeds Hospital Magazine, Provincial Medical Journal, Homœopathic Review, Toilers of the Deep, Our Waifs and Strays, Nursing Notes, The News, Child's Guardian, "Number One's" Book, by Edward Caulfield Houston; Science Progress, Service for the King, The Practitioner, Bulletin of Johns Hopkins' Hospital, Journal d'Hygiène, Engineering Record, Journal de Médecine, Progrès Médical, Publisher's Circular, Rural World, The Planter, Public Health, The Sun, The Medical Pioneer, Charity Organization Review, Great Thoughts, Chicago Clinical Review.

### THE STUDENT OF MEDICINE.

### APPOINTMENTS.

W. C. Allardice, M.B., C.M.Glasg., appointed House Physician to the North Staffordshire Infirmary and Eye Hospital, Stoke-upon-Trent. J. J. Cowan, M.B.Edin., appointed Medical Officer for the No. 3 District of the Bromyard Union. H. Downes, L.R.C.P.E., L.R.C.S.E., L.F.P.S.Glasg., appointed Assistant House Surgeon to Branch Hospital, Seamen's Hospital Society, Victoria and Albert Docks. V. J. Hodgson, L.R.C.P.Lond., M.R.C.S.Eng., D.P.H.Cantab., reappointed Assistant Medical Officer of Health to the Port of London. P. Liston, L.R.C.P., L.R.C.S.I., appointed Medical Officer of Balrothery Workhouse, Lusk, co. Dublin; and also Consulting Medical Officer of Health for Balrothery Union. H. W. Maclure, M.B., B.S., B.A.Cantab., M.R.C.S.Eng., L.R.C.P.Lond., appointed Medical Officer to the Metropolitan Fire Brigade, District No. 6, by the London County Council. L. G. S. Molloy, M.D.Dublin, appointed Surgeon (for one year) to Blackpool Hospital. M. O'Driscoll, L.M.I., appointed Medical Officer for the Union Hall Dispensary District. T. A. Palm, M.D.Edin., reappointed Medical Officer of Health to the Wigton Local Board. W. Roydon, L.R.C.P.Lond., M.R.C.S. Eng., appointed Medical Officer of Health to the Rural Sanitary District of the East and West Incorporation. A. S. Taylor, B.A., B.C., M.D.Cantab., F.R.C.S,Eng., appointed Honorary Medical Officer to the Surbiton Cottage Hospital. J. A. T. White, L.R.C.P.Lond., M.R.C.S.Eng., appointed Medical Officer for the Hatfield District of the Dunmow Union. M. Young, M.B., C.M.Edin., Medical Officer of Health to the Halifax Rural Sanitary Authority, appointed Medical Officer of Health to the Corporation of Brighouse, Yorks.

### VACANCIES.

**Resident Medical Officers.**

Aberdeen City Hospital.—Resident Physician at the City (Infectious Diseases) Hospital. Salary £50 per annum, with board and lodgings at the Hospital. Applications to W. Gordon, Town Clerk, Town House, Aberdeen, by August 27th.

County Asylum, Prestwich, Manchester.—Pathologist. Salary £200 per annum, with board, residence, and washing. Applications and testimonials to the Superintendent.

Cumberland Infirmary, Carlisle.—House Surgeon. Salary £70 per annum, with board, lodging, and washing. Applications and testimonials to the Secretary before August 29th.

General Hospital, Nottingham.—Senior Resident Medical Officer; doubly qualified. Salary £120 for the first year, with an addition of £10 a year up to £150, with board, residence, and washing. Applications to the Secretary by September 1st.

Memorial Hospital, Jarrow-on-Tyne.—House Surgeon, age not under 25, and to reside and board free in the hospital. To come under a three years' engagement at a progressive salary of £130, £150, and £170 respectively. Applications and testimonials to J. Campbell, Secretary, by September 4th.

Miller Hospital and Royal Kent Dispensary, Greenwich Road, S.E.—Junior Resident Medical Officer, qualified. Salary £30 per annum, with board, attendance, and washing; tenable for six months, with prospect of re-election as Senior. Salary £60 per annum. Applications and testimonials to Major-General G. R. Roberts, Honorary Secretary, by August 31st.

North Dispensary, Liverpool.—Head Surgeon. Salary £200 per annum, with apartments, board, and attendance. Applications and testimonials to R. R. Greene, Secretary, Leith Offices, Moorfields, Liverpool, by August 27th.

Royal Orthopædic Hospital, 297, Oxford Street.—Resident House Surgeon. Candidates must be M.R.C.S.Eng. and L.R.C.P., unmarried. Salary £100 per annum, with residence and partial board. Applications and testimonials to the Secretary by August 28th.

St. Peter's Hospital, Henrietta Street, Covent Garden, W.C.—House Surgeon for six months. Salary at the rate of 50 guineas a year, with board, lodging, and washing, and an allowance for wine, &c. Applications and testimonials to Irwin H. Beattie, Secretary, before September 1st.

South Dispensary, Liverpool.—Head Surgeon. Salary £200 per annum, with apartments, board, and attendance. Applications and testimonials to R. R. Greene, Secretary, Leith Offices, Moorfields, Liverpool, by August 27th.

South Dispensary, Liverpool.—Assistant Surgeon. Salary £80 the first year, and £90 per annum afterwards, with apartments, board, and attendance. Applications and testimonials to R. R. Greene, Secretary, Leith Offices, Moorfields, Liverpool, by August 27th.

THE STUDENT OF MEDICINE—*continued.*

**Non-Resident Medical Officers.**

Aberdeen Royal Infirmary.—Ophthalmic Surgeon. Applications by August 31st.

Burgh of Leith.—Medical Officer of Health. Salary to commence at £350. Applications and testimonials to T. B. Laing, Town Clerk's Office, Leith, by September 1st.

COUNTY ASYLUM, PRESTWICH, MANCHESTER.

ST. PETER'S HOSPITAL, HENRIETTA STREET, COVENT GARDEN, W.C.

